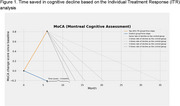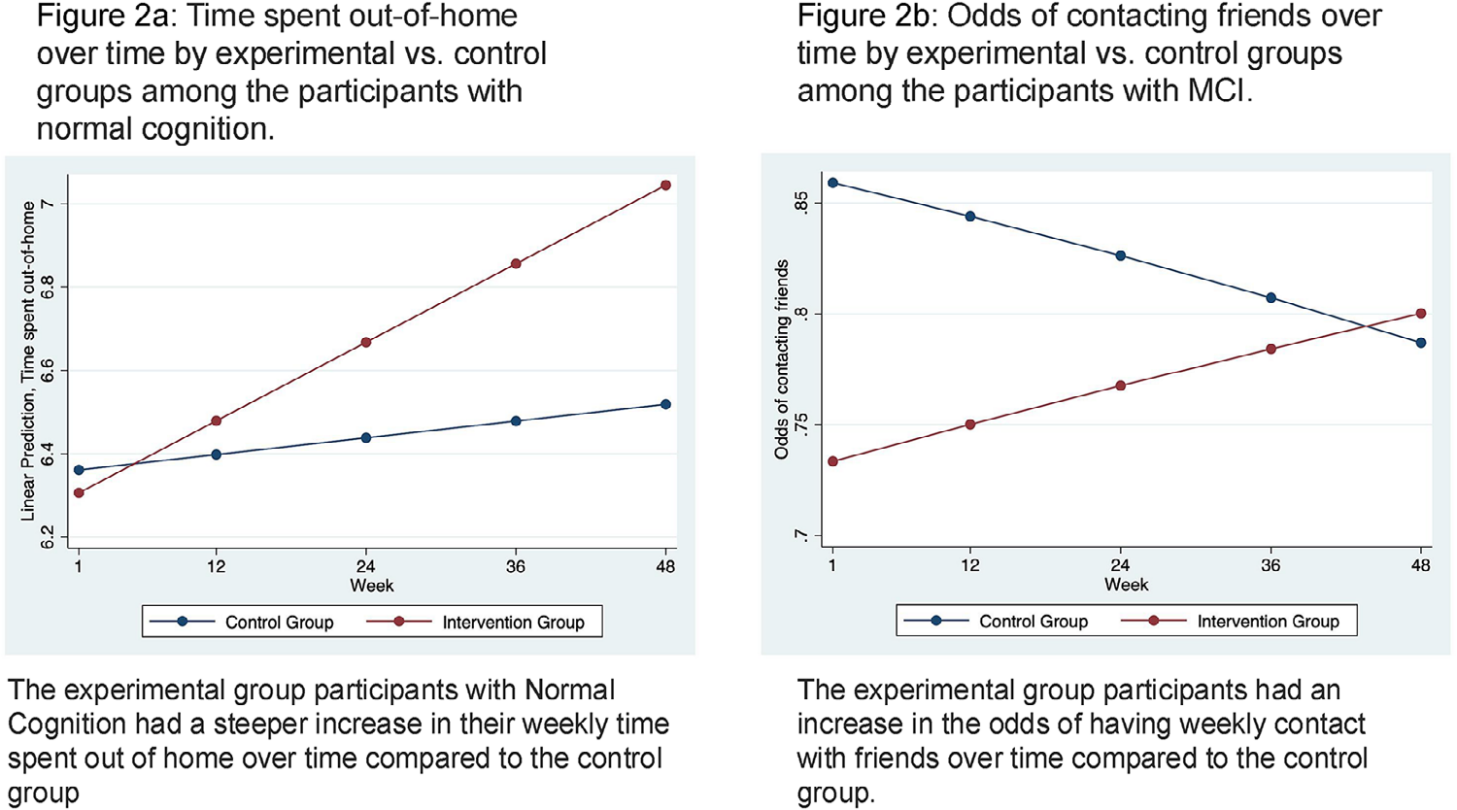# Internet‐Based Conversational Engagement Clinical Trial (I‐CONECT): Overview and new findings

**DOI:** 10.1002/alz70860_100904

**Published:** 2025-12-23

**Authors:** Hiroko H Dodge, Chao‐Yi Wu, Liu Chen, Kexin Yu

**Affiliations:** ^1^ Massachusetts General Hospital, Harvard Medical School, Boston, MA, USA; ^2^ University of Texas Southwestern Medical Center, Dallas, TX, USA

## Abstract

**Background:**

Growing evidence identifies social isolation as a risk factor for dementia. Addressing social isolation to delay cognitive decline could help reduce dementia prevalence. The Internet‐based Conversational Engagement Clinical Trial (I‐CONECT; www.i‐conect.org; ClinicalTrials.gov: NCT02871921, *N* = 186) recruited socially isolated older adults with normal cognition or mild cognitive impairment (MCI), providing frequent conversational interactions with trained interviewers. Topline results previously demonstrated its efficacy on cognitive functions and brain connectivity. Here, we present two new findings on the clinical relevance of this intervention: (1) time saved in delaying cognitive decline (time‐saved analysis) and (2) the intervention's impact on behavioral changes in daily life.

**Methods:**

(1) Time‐saved analysis: Individual treatment response (ITR) analyses identified participants who benefited most from the intervention, estimating the months of delayed cognitive decline compared to the control group. (2) Intervention effect on daily social interactions: Weekly telephone surveys (up to 12 months) tracked the frequency and duration of social contact with family and friends, as well as time spent out of the house (a proxy for social interaction). Mixed‐effects models analyzed these trajectories.

**Results:**

(1) The top 30% of the responders could delay the cognitive decline by 6 months or more in the global cognitive function (Figure 1). (2) Among those with normal cognition, the experimental group had a steeper increase in their time spent out‐of‐home (B = 0.012, SE = 0.005, *p* = 0.015). The MCI experimental group participants had an increased likelihood of contacting friends over time compared to the control group with the significant interaction of the time (week) and intervention group (interaction B=1.031, SE=0.009, *p* = 0.001) (Figure 2). The intervention did not influence participants’ social contact with family members.

**Conclusions:**

The responders to the intervention could delay the cognitive decline by 6 months, even with conservative estimates. The weekly social interaction data suggest that I‐CONECT intervention can activate weekly activities among socially isolated older adults, which could bring additional cognitive stimulations and prevent cognitive decline. These results further add evidence of the beneficial effects of social interactions among socially isolated older subjects and solidify the previous findings.